# Macrophage induces anti-cancer drug resistance in canine mammary gland tumor spheroid

**DOI:** 10.1038/s41598-023-37311-w

**Published:** 2023-06-27

**Authors:** Ga-Hyun Lim, Ju-Hyun An, Su-Min Park, Ga-Hee Youn, Ye-In Oh, Kyoung-Won Seo, Hwa-Young Youn

**Affiliations:** 1 Laboratory of Veterinary Internal Medicine, Department of Veterinary Clinical Science, College of Veterinary Medicine, Seoul National University, Seoul, 08826 Republic of Korea; 2 Department of Veterinary Emergency and Critical Care Medicine, Institute of Veterinary Science, College of Veterinary Medicine, Kangwon National University, Chuncheon-si, Republic of Korea; 3 Department of Veterinary Internal Medicine, College of Veterinary Medicine, Kyungpook National University, Daegu, Republic of Korea

**Keywords:** Cancer, Cell biology, Immunology, Molecular biology

## Abstract

Tumor-associated macrophages (TAMs) play an important role in the tumor microenvironment by producing cytokines and growth factors. Furthermore, TAMs play multifunctional roles in tumor progression, immune regulation, metastasis, angiogenesis, and chemoresistance. Hypoxia in the tumor microenvironment induces tumor-supporting transformation of TAMs, which enhances tumor malignancy through developing anti-cancer resistance, for example. In this study, a hybrid spheroid model of canine mammary gland tumor (MGT) cell lines (CIPp and CIPm) and canine macrophages (DH82) was established. The effects of hypoxia induced by the spheroid culture system on the anti-cancer drug resistance of canine MGT cells were investigated. A hybrid spheroid was created using an ultralow-adhesion plate. The interactions between canine MGT cells and DH82 were investigated using a co-culture method. When co-cultured with DH82, cell viability and expression levels of tumor growth factors and multi-drug resistance genes were increased in canine MGT cells under doxorubicin. Additionally, doxorubicin-induced apoptosis and G2/M cell cycle arrest were attenuated in canine MGT cells co-cultured with DH82. In conclusion, the hybrid spheroid model established in this study reflects the hypoxic TME, allowing DH82 to induce anti-cancer drug resistance in canine MGT cells.

## Introduction

Mammary tumors are one of the most common tumors in both human women and female canines^1–3^. In female canines, more than 40% of tumors are mammary gland tumors (MGT), 30–50% of which are malignant^3–6^. Surgery is considered the primary treatment for canine MGT, but several cases are accompanied by micrometastases at the time of surgery^3,7,8^. Similarly, in humans, micrometastases can be present at the time of surgery^9^; thus, adjuvant treatments are applied to lower the possibility of recurrence and metastasis in both women and female canines^10^. However, it has not yet been elucidated whether the application of adjuvant chemotherapy in canine MGTs has a significant therapeutic effect^11^. Anti-cancer drug resistance is a major problem in chemotherapy for mammary tumors^12,13^. Several studies have been conducted on mammary tumors resistance; in particular, the tumor microenvironment (TME) is considered the key mechanism of anti-cancer drug resistance^12,14–16^.

Macrophages that infiltrate tumors and constitute the TME are known as tumor-associated macrophages (TAM). TAMs play a major role in tumor growth, angiogenesis, metastasis, immunoregulation, and anti-cancer drug resistance in the TME^15–19^. Macrophages are traditionally classified as M1 or M2 phenotype. M1 polarized TAMs possess a proinflammatory response and anti-tumor effect, whereas M2 polarized TAMs possess an anti-inflammatory response and pro-tumor effect^12,17–20^. Microenvironmental signals, such as colony stimulating factor (CSF)-1 ad CCL-2 secreted by tumor cells or stromal cells, regulated TAM polarization^19,21,22^. TAMs can constitute up to 50% of the tumor mass, and most TAMs are M2 polarized^23^. Several studies have revealed that TAMs induce tumor chemoresistance through various mechanisms, and mammary tumors reduce the therapeutic response to anti-cancer drugs through mechanisms, such as IL-10/STAT3/Bcl2 signaling^12,24,25^.

Therefore, several studies have used TAMs as a novel target in anti-cancer therapy. However, conventional 2D culture methods have limitations in reflecting TAM containing TME. Therefore, a 3D structural culture method that mimics the TME, such as cell–cell interaction, nutrient and oxygen demands, and drug penetration, is required to investigate the role of TAMs^26,27^. In large spheroids (~ 500 μm), hypoxia and malnutrition of the core induce necrosis, which is analogous to the in vivo hypoxic microtumor known to contribute to anti-cancer drug resistance^26,28^. Hypoxia affects macrophage polarization and influences the crosstalk between tumor cells and TAMs in the TME^29^. Therefore, 3D spheroid culture is considered a novel tool for evaluating the interactions between TAMs and tumor cells^30,31^.

In this study, we constructed a 3D multicellular hybrid spheroid model of canine MGT cells with DH82. We confirmed the effects of hypoxia on the growth factors and multi-drug resistance of canine MGT cells in the spheroid model. Moreover, infiltration of DH82 in the hypoxia spheroid model was identified. Finally, we confirmed that DH82 induces anti-cancer drug resistance by examining the effects of doxorubicin on growth, apoptosis, cell cycle arrest, and multi-drug resistance gene expression in canine MGT cells. Therefore, the ultimate objective is to investigate whether a hybrid tumor spheroid model with macrophages is suitable for anti-cancer drug research.

## Results

### Canine mammary gland tumor hybrid spheroid with macrophages

#### Construction of hybrid spheroid model and characterization

**Figure 1:**
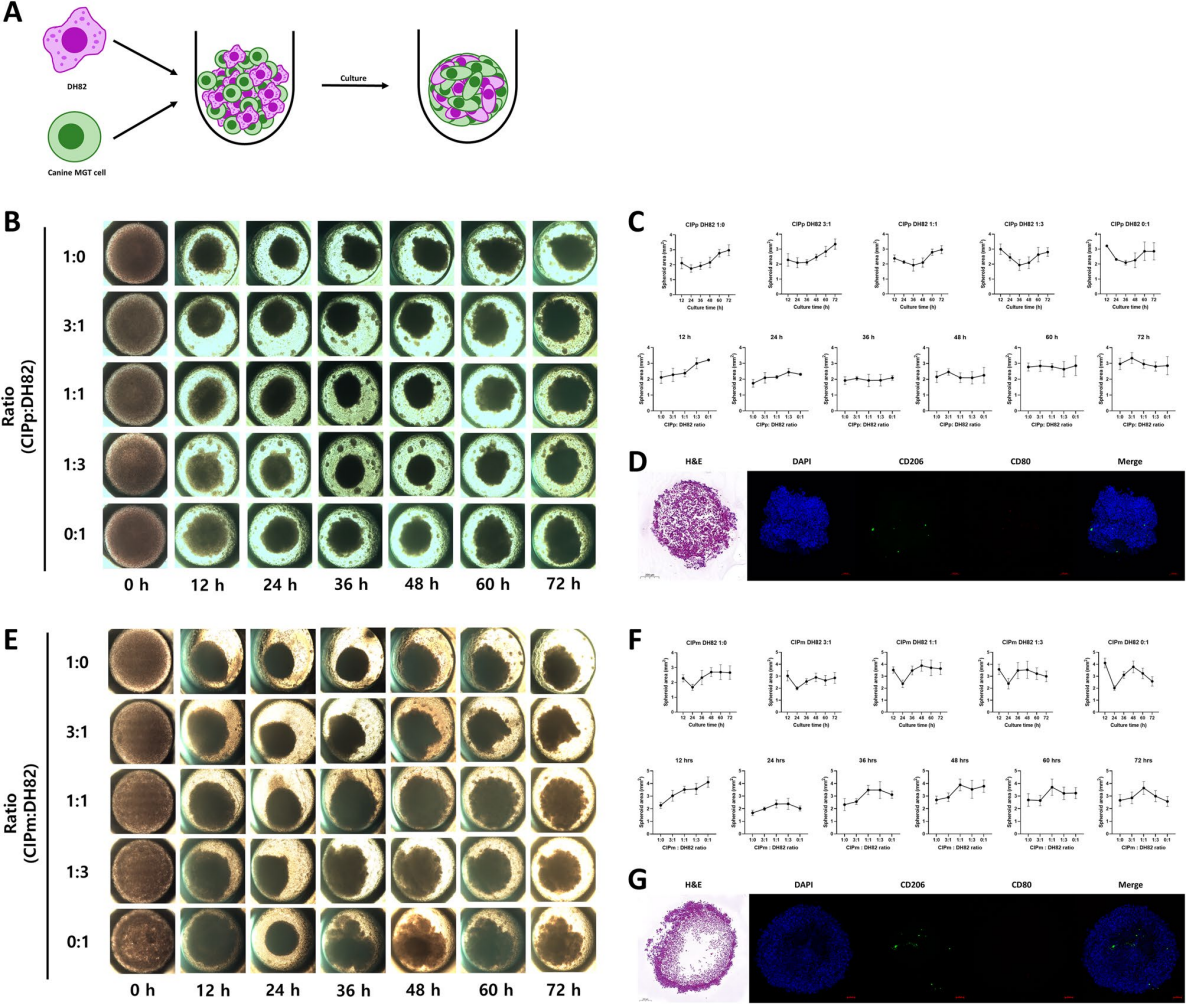
Establishment of hybrid spheroid formation conditions and histological examination of hybrid spheroid. (**A**) Method of hybrid spheroid formation. Canine MGT cells and DH82 were seeded in an ultra-low adhesion plate at a set ratio. Mixed cells were cultured to form an appropriate spherical shape and then used in experiments. The top panels (**B–D**) are CIPp and the bottom panels (**E–G**) are CIPm. (**B,E**) Hybrid spheroids shape according to culture time and ratio of canine MGT cells and macrophages. (**C,F**) Hybrid spheroids size according to culture time and ratio of canine MGT cells and macrophages. (**D,G**) H&E staining images of hybrid spheroid (canine MGT cell:DH82 = 3:1) sections (grey bar = 200 μm) and Confocal images of hybrid spheroids (red bar = 100 μm). Anti-CD206 (green) and anti-CD80 (red) were used to confirm polarization of DH82 (blue = nuclei DAPI staining). The results are presented as the mean ± SD of triplicate samples and are representative of 3 independent experiments. MGT, mammary gland tumor; H&E, hematoxylin, and eosin; DAPI, 4,6-diamidino-2-phenylindole.

Canine MGT cells and DH82 cells were cultured on ultra-low adhesion plates for 3 days with different ratios of canine MGT cells and DH82 (1:0, 3:1, 1:1, 1:3, and 0:1) (Fig. 1A). To determine the optimal culture ratio and time, the area of the hybrid spheroids was measured every 12 h (Fig. 1B, E). We measured the area of spheroids for 72 h; for CIPp hybrid spheroids, it was confirmed that the most condensation occurred at a ratio of 1:1 for culturing 36 h (1.929 ± 0.3943 mm^2^) (Fig. 1C). CIPm hybrid spheroids were the most condensed when incubated for 24 h at a ratio of 3:1 (1.990 ± 0.1362 mm^2^) (Fig. 1F). To confirm the interaction between canine MGT cells and DH82, CIPp and CIPm hybrid spheroids were cultured for 36 h at a ratio of 3:1. To evaluate the degree of aggregation between cells in hybrid spheroids, the spheroid was sectioned, and performed hematoxylin and eosin (H&E) staining. In CIPm hybrid spheroids, the degree of cell aggregation in the core was lower than that in CIPp hybrid spheroids (Fig. 1D, G). Immunofluorescence staining for CD206 and CD80 was performed to evaluate macrophage polarization. It was confirmed that M1/M2 polarized DH82 were distributed in both hybrid spheroids (Fig. 1D, G).

#### Increased COX2, HIF-1α, growth factors and multi-drug resistance gene expression in canine MGT cells alone spheroids

**Figure 2:**
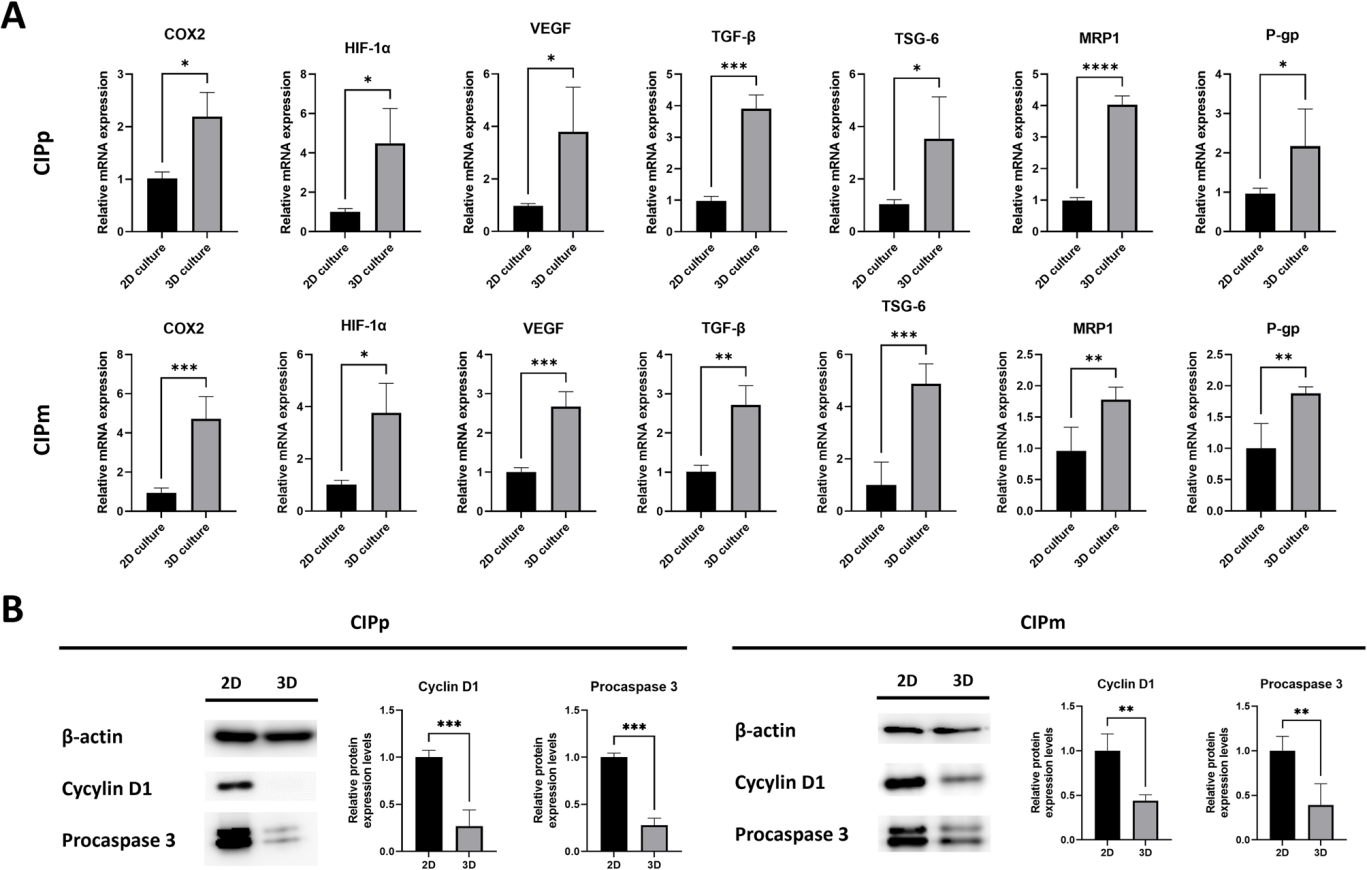
3D culture condition increase expression levels of growth factors and multi-drug resistance genes in canine MGT cells and affect apoptosis and cell cycle arrest in canine MGT cells. (**A**) mRNA relative expression of growth factors and multi-drug resistance genes in canine MGT cells cultured as 2D or 3D spheroid for 48 h. (**B**) Cyclin D1 and procaspase 3 protein expression in canine MGT cells cultured as 2D or 3D spheroid for 48 h. The results are presented as the mean ± SD of triplicate samples of three independent experiments. *MGT* mammary gland tumor, *COX2* cyclooxygenase 2, *HIF-1α* hypoxia-inducible factor 1-alpha, *VEGF* vascular endothelial growth factor, *TGF-β* transforming growth factor β, *TSG-6* TNF-stimulated gene 6 protein, *MRP1* multi-drug resistance-related protein, *P-gp* P-glycoprotein, *2D* 2-dimensional, *3D* 3-dimensional. *p < 0.05, **p < 0.01, ***p < 0.001, ****p < 0.0001, as determined by Student's t-tests.

Unlike 2D culture, hypoxia is occurred in the core of spheroids^32,33^. In the TME, hypoxia induces tumor growth, angiogenesis, and M2 macrophage polarization, affecting anti-cancer drug resistance^16^. Therefore, we attempted to identify whether hypoxia affects the expression levels of COX2, HIF-1α, growth factors and multidrug resistance genes in canine MGT cultured as spheroids. Canine MGT cells were cultured in 2D cell culture plates and Stem FIT 3D cell culture dishes for 48 h at 37 °C. In both CIPp and CIPm, the expression levels of COX2, HIF-1α, and growth factors were significantly increased in both CIP alone spheroids (Fig. 2A). Additionally, the expression levels of multidrug resistance genes such as P-gp and MRP1 were significantly increased in canine MGT cells cultured in CIP alone spheroids (Fig. 2A).

#### Hypoxia effects to cell cycle distribution and apoptosis in canine MGT cells alone spheroids

The hypoxic environment in 3D culture affects cell cycle distribution and apoptosis^32,34–36^. To confirm whether cells were exposed to hypoxia induced by the spheroid culture system, the expression levels of cell cycle- and apoptosis-related proteins were evaluated. In both CIP alone spheroids, Cyclin D1, which is associated with the cell cycle, was downregulated (Fig. 2B). Moreover, the expression level of procaspase 3 was significantly decreased in both CIP alone spheroids (Fig. 2B).

### Anti-cancer drug resistance induced by macrophages in canine mammary gland tumor cells

#### Cell viability of canine MGT cells and macrophages under doxorubicin (DOX)

**Figure 3:**
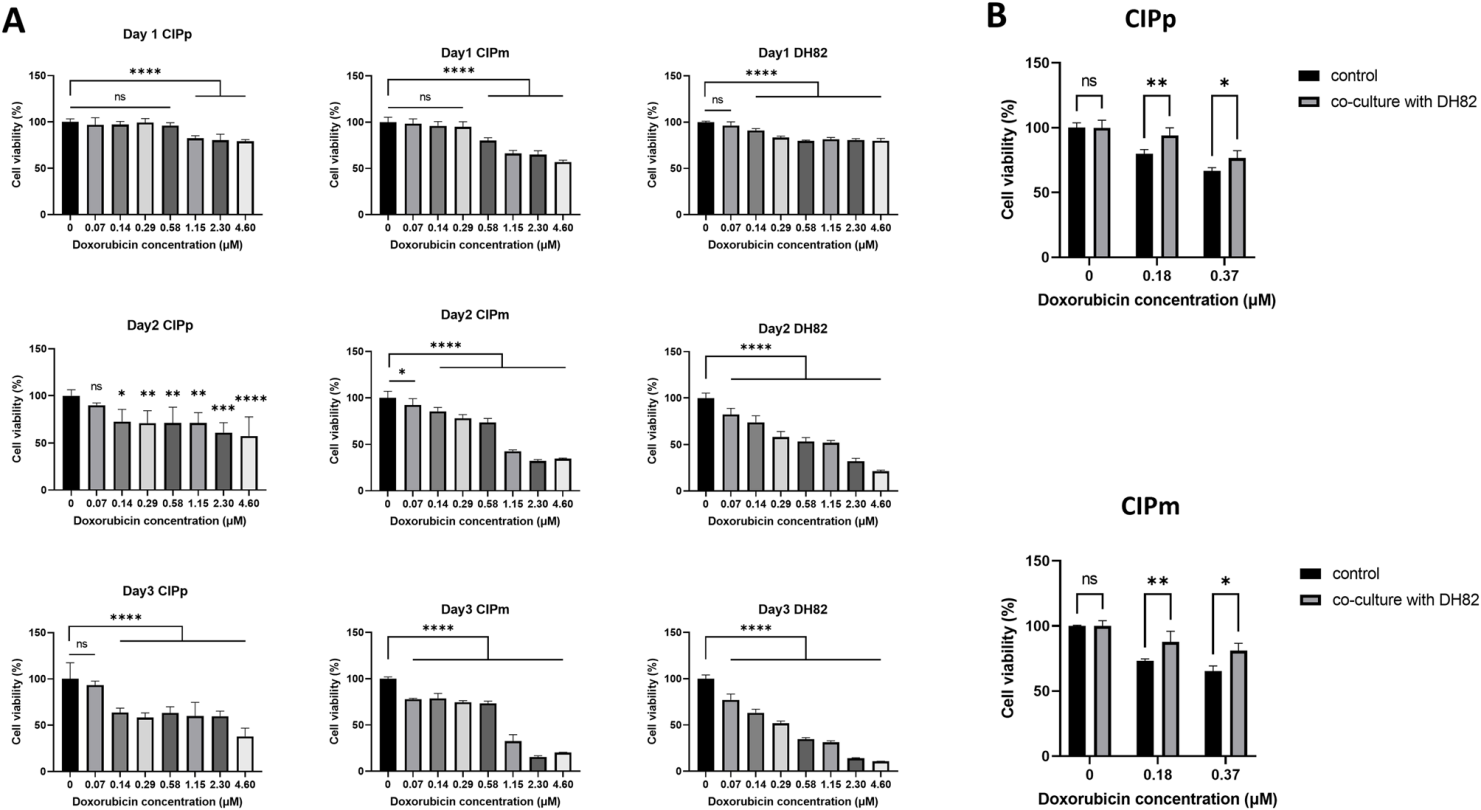
Cell viability of canine MGT cells and DH82 under doxorubicin. (**A**) Doxorubicin inhibited proliferation of CIPp, CIPm, and DH82 in a dose-dependent manner. (**B**) Cell viability results in canine MGT cells co-cultured with DH82 under doxorubicin (0, 0.18, and 0.37 μM) for 48 h. The cytotoxicity of doxorubicin was reduced in co-cultured canine MGT cells compared to controls. The results are presented as the mean ± SD of triplicate samples of 3 independent experiments. *TC* tumor cell, *2D* 2-dimensional, *3D* 3-dimensional, *ANOVA* analysis of variance. *p < 0.05, **p < 0.01, ***p < 0.001, ****p < 0.0001, as determined by one-way ANOVA and two-way ANOVA.

To evaluate the cytotoxic effect of DOX, canine MGT and DH82 cells were treated with different concentrations of DOX, and cell viability was analyzed using the Cell Counting Kit-8 (CCK-8) assay at 24, 48, and 72 h. The viability of canine MGT and DH82 cells decreased in a dose-dependent manner (Fig. 3A). To evaluate anti-cancer drug resistance, we set the DOX concentration at which cell viability was significantly reduced. It was confirmed that the cell viability was significantly reduced at concentrations of 0.14 μM or higher at 48 h.

#### Macrophages induce anti-cancer drug resistance in canine MGT cells

To confirm the anti-cancer drug resistance affected by DH82, cell viability was measured at concentrations of 0.18 and 0.37 μM DOX at 48 h in canine MGT cells with co-culture method. As shown in Fig. 3B, cell viability increased significantly in canine MGT cells co-cultured with DH82 compared to canine MGT cells alone.

### Effect of macrophage on canine mammary gland tumor cells under anti-cancer drug

#### Macrophages induce hypoxia and increase anti-cancer resistance related gene expression in canine MGT cells

**Figure 4:**
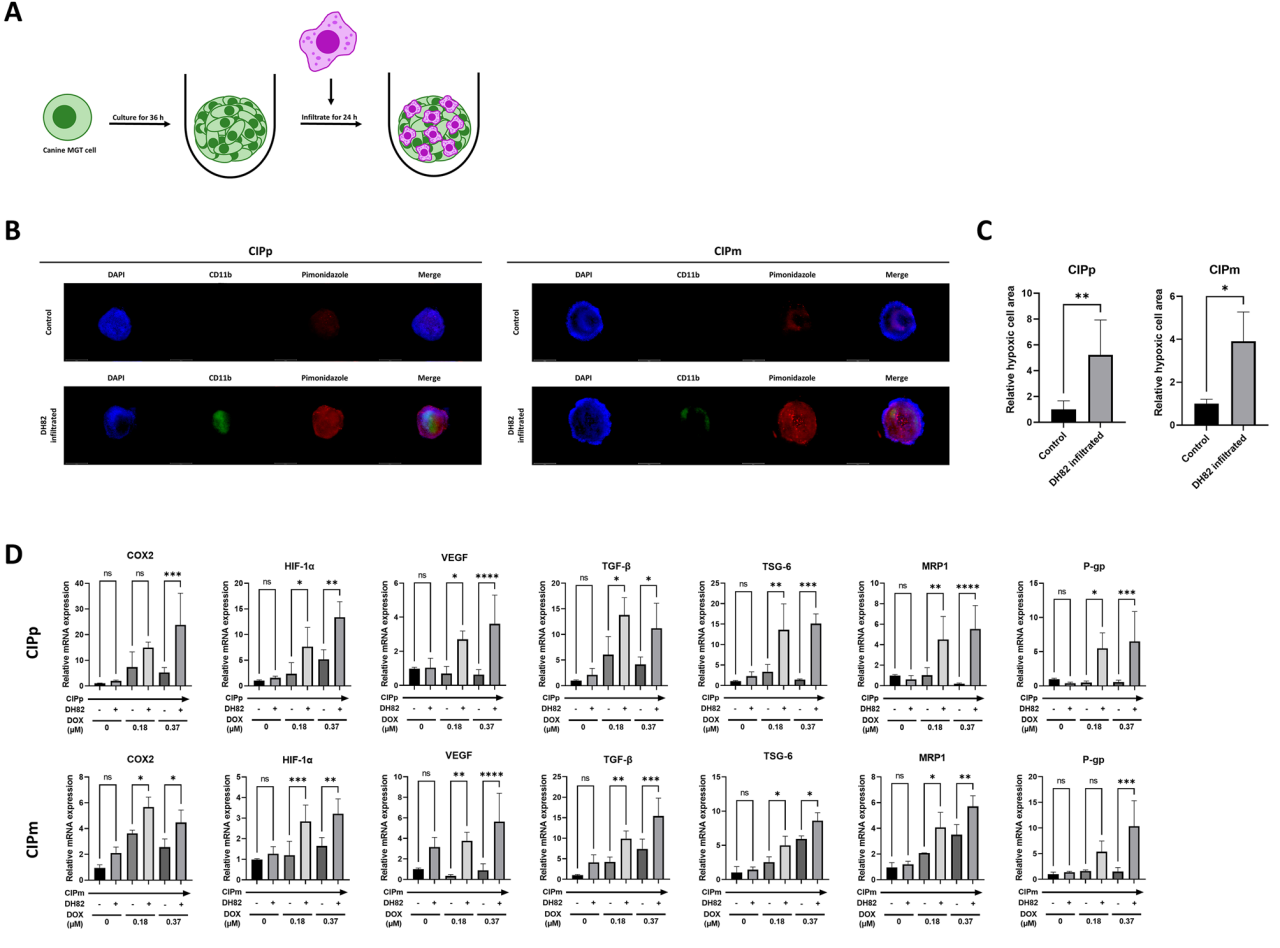
DH82 induces hypoxia in canine MGT cells and affects tumor cell growth factor and multi-drug resistance gene expression under doxorubicin. (**A**) DH82 infiltration model using CIP alone spheroid. Canine MGT cells were seeded in an ultra-low adhesion plate alone. After spheroid formed, DH82 were added into the well and co-culture for 24 h to infiltrate the spheroid. (**B**) Immunofluorescence imaging of DH82 infiltrated spheroids. 500 μM pimonidazole was treated for 2 h before fixation. Spheroids were stained with DAPI (blue), FITC dye -conjugated CD11b mouse monoclonal antibody (green) and APC dye -conjugated IgG_1_ mouse monoclonal anti-pimonidazole antibody (red) (white bar = 650 μm). (**C**) Relative hypoxic cell area of spheroids with or without DH82 infiltration. (**D**) mRNA relative expression of growth factors and multi-drug resistance genes in canine MGT cells co-cultured with or without DH82 under doxorubicin (0, 0.18, and 0.37 μM) for 48 h. The results are presented as the mean ± SD of triplicate samples of three independent experiments. *MGT* mammary gland tumor, *COX2* cyclooxygenase 2, *HIF-1α* hypoxia-inducible factor 1-alpha, *VEGF* vascular endothelial growth factor, *TGF-β* transforming growth factor β, *TSG-6* TNF-stimulated gene 6 protein, *MRP1* multi-drug resistance-related protein, *P-gp* P-glycoprotein, *DAPI* 4,6-diamidino-2-phenylindole, *APC* allophycocyanin, *IgG1* immunoglobulin G1. *p < 0.05, **p < 0.01, ***p < 0.001, ****p < 0.0001, as determined by one-way ANOVA.

We tried to confirm whether the hypoxic status of canine MGT cells is affected by DH82. DH82 cells were infiltrated to the spheroids of canine MGT cells, cultured for 24 h (Fig. 4A), and the hypoxic status was evaluated using pimonidazole. The hypoxic cells increased in DH82-infiltrated spheroids (Fig. 4B, C).

We also analyzed the expression of COX2, HIF-1α, growth factors and multi-drug resistance genes in canine MGT cells by DH82 co-culture method. The expression levels of COX2, HIF-1α, growth factors and multidrug resistance genes were analyzed in canine MGT cells co-cultured with DH82. Under DOX treatment, when co-cultured with DH82, COX2, HIF-1α, growth factors and multidrug resistance gene expression increased in canine MGT cells (Fig. 4D).

#### Macrophages reduce apoptosis induced by doxorubicin in canine MGT cells

**Figure 5:**
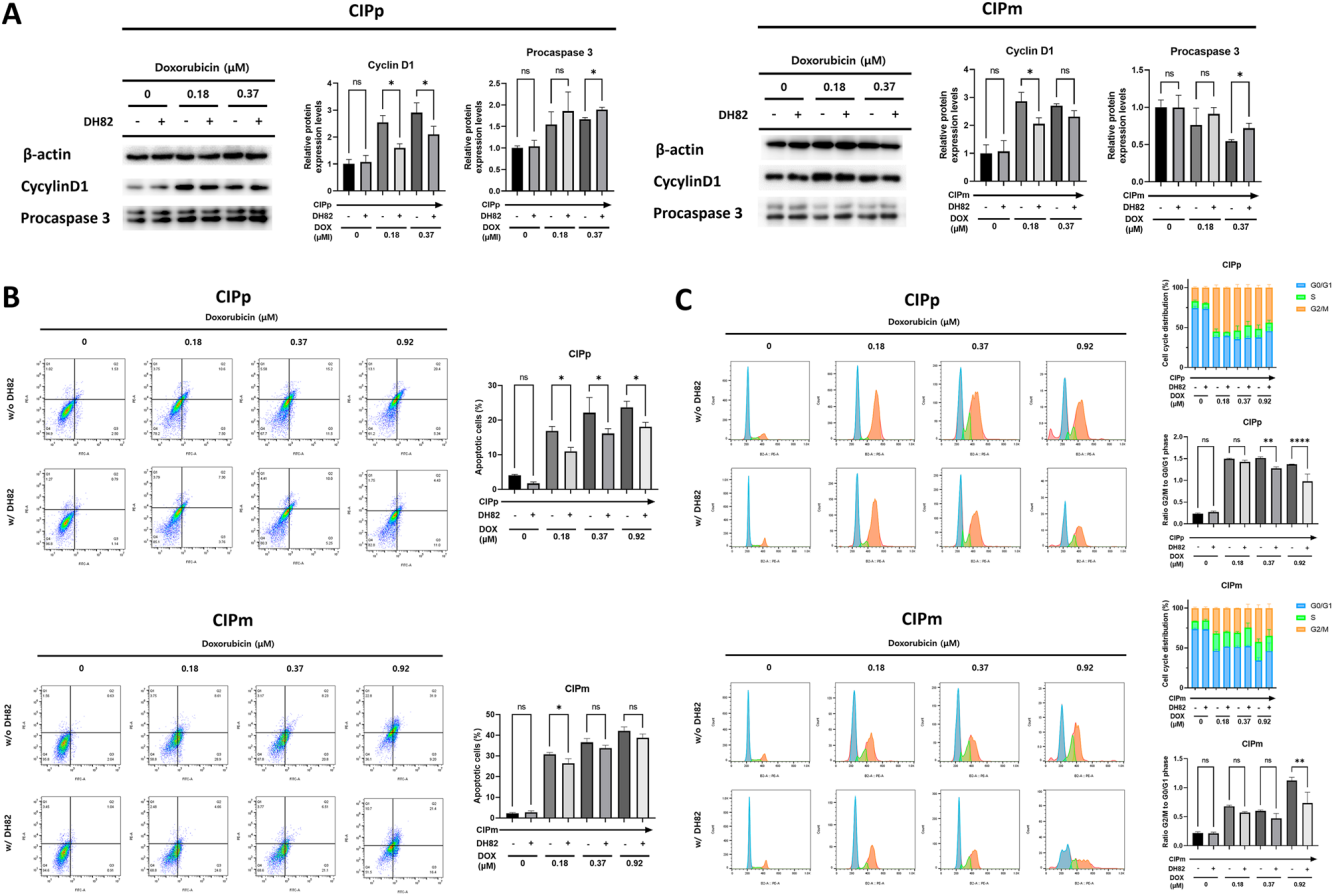
DH82 reduces apoptosis and G2/M cell cycle arrest in canine MGT cells under doxorubicin. Canine MGT cells co-cultured with or without DH82 under doxorubicin (0, 0.18, 0.37, and 0.92 μM) for 48 h. (**A**) Protein expression of cyclin D1 and procaspase 3 in canine MGT cells measured by western blotting. Cyclin D1 and procaspase 3 band density was estimated using Image J and normalized to that of β-actin. (**B**) The percentage of apoptotic ratio of canine MGT cells was measured by flow cytometry using Annexin V-FITC and PI staining. Early apoptotic cells were stained as Annexin-V+ /PI−. Late apoptotic cells were stained as Annexin V+/PI+. (**C**) The cell cycle distribution of canine MGT cells was measured by flow cytometry using PI staining. The percentage of canine MGT cells in G0/G1, S and G2/M are shown. The results are presented as the mean ± SD of triplicate samples of three independent experiments. *MGT* mammary gland tumor, *FITC* fluorescein isothiocyanate, *PI* propidium iodide. *p < 0.05, **p < 0.01, ****p < 0.0001, as determined by one-way ANOVA.

To evaluate the mechanism by which DH82 regulate anti-cancer drug-induced apoptosis in canine MGT cells, procaspase 3 protein expression levels and the ratio of apoptosis were analyzed using annexin-V/PI staining. Under 0.37 μM DOX, procaspase 3 was upregulated in canine MGT cells co-cultured with DH82 compared to canine MGT cells alone. (Fig. 5A). Additionally, in co-cultured CIPp at 0.18, 0.37, and 0.92 μM DOX and co-cultured CIPm at 0.18 μM DOX, annexin-V positive apoptotic cells were significantly reduced compared to canine MGT cells alone group at the same DOX concentrations (Fig. 5B).

#### Macrophages reduce G2/M phase cell cycle arrest induced by doxorubicin in canine MGT cells

To investigate the effect of DH82 on the cell cycle arrest of canine MGT cells treated with DOX, Cyclin D1 protein expression was measured using western blotting. When compared to control, cyclin D1 was downregulated in co-cultured CIPp at 0.18 and 0.37 μM DOX and co-cultured CIPm at 0.18 μM DOX (Fig. 5A). The cell cycle distribution of canine MGT cells was analyzed using PI staining. The CIPp and CIPm control groups had the highest proportion of cells in the G0/G1 phase. Compared with the control group, the percentage of cells in the G2/M phase increased when treated with DOX, whereas the percentage of cells in the G0/G1 phase decreased. The G2/M phase arrest was reduced in co-cultured CIPp at 0.37, 0.37 and 0.92 μM DOX and co-cultured CIPm at 0.92 μM DOX (Fig. 5C).

## Discussion

In this study, a hybrid spheroid model was established by mixing canine MGT cells with DH82 to confirm the function of macrophages in inducing anti-cancer drug resistance in canine MGT cells. As in previous canine lymphoma spheroid model studies, 3D culture was performed using an ultralow adhesion plate^37^. First, the size and sphericity of spheroids were evaluated according to the timings and ratios of canine MGT cells and DH82 to corroborate the parameters under which spheroids are generated. In setting the spheroid formation conditions, the appropriate condition for forming the spherical shape was chosen while determining the spheroid formation circumstances. H&E staining of the spheroid sections confirmed that the cells were not densely packed into the core. The decrease in cell density of the spheroid and core is considered due to the apoptosis and necrosis caused by a decrease in oxygen and nutrients in the inner cells of the spheroid^28,32,34^.

Hypoxia is one of the hallmarks of cancer and affects several aspects of tumorigenesis, including proliferation, angiogenesis, immunosurveillance, metabolism, and malignancy^9–11^. HIF-1a is a key transcription factor induced by hypoxia and induces the production of growth factors, including VEGF, and TGFβ^10–12^. We attempted to determine the effect of hypoxia on canine MGT cells cultured in CIP alone spheroids. First, the expression of COX2, HIF-1a and growth factors, including VEGF, and TGFβ increased in canine MGT cells cultured in CIP alone spheroids. HIF-1a contributes to hypoxia-induced drug resistance by activating multidrug resistance gene expression^14^. Regarding this, we confirmed the upregulation of MRP1 and P-gp in canine MGT cells of CIP alone spheroids. Moreover, apoptosis and cell cycle changes have been detected in other spheroid models^38–40^. Thus, we examined apoptosis and cell cycle arrest in CIP alone spheroids by analyzing procaspase 3 and cyclin D1 expression levels. In our results, procaspase 3 was decreased, which is associated with increased apoptosis in canine MGT cells in CIP alone spheroids. This is considered to be related to the activation of p53 dependent apoptosis by HIF-1a^41,42^. Cyclin D1, which regulates the G1-S phase transition, was downregulated in canine MGT cells of CIP alone spheroids. In relation to that, HIF-1 regulates cyclin D1 expression by binding to its promoter region of cyclin D1^43,44^. Taken together, these results suggest that the spheroid culture method mimic the TME by creating a hypoxic environment, similar to other 3D culture models known to reflect the TME^28,32^.

TAM migrate to the hypoxic area of tumors and contribute to hypoxia in tumor cells^45^. Hypoxia induces M2 polarization of TAM in the TME^17^. To confirm the polarization of macrophages in the hybrid spheroid model, the expression of CD206 and CD80 was analyzed. Confocal imaging confirmed that the M1 and M2 macrophages were distributed within the hybrid spheroids. Previous studies have shown that TSG-6 induces M2 polarization by affecting COX2 expression, and HIF-1a is known to affect TSG-6 expression^46,47^. In this study, we confirmed the expression levels of HIF-1a, TSG-6, COX2, and VEGF increased in canine MGT cells in CIP alone spheroids. In addition, hypoxic cells were increased in the spheroid infiltrated with DH82. Therefore, we considered that the hybrid spheroid model mimics the hypoxic TME by reflecting the cross talk between canine MGT cells and DH82.

TAM contributes to anti-cancer drug resistance in tumors^12,17,24^. To evaluate the anti-cancer drug resistance effects of macrophages, we investigated the expression levels of COX2, HIF-1α, growth factors and multidrug resistance genes, cell viability, apoptosis rates, and cell cycle arrest in canine MGT cells co-cultured with DH82^48,49^. In 2D co-culture method, the viability of canine MGT cells co-cultured with DH82 under doxorubicin was increased compared to control. Furthermore, we confirmed that HIF-1α, MRP1, and P-gp were upregulated in canine MGT cells co-cultured with DH82 under doxorubicin treatment. Given that cross talk of canine MGT cells and DH82 is associated with hypoxia, it is considered that HIF-1a contributes to the expression of MRP1 and P-gp to induce drug efflux in canine MGT cells, resulting in anti-cancer drug resistance. Expression of VEGF^1^, COX2^50,51^, TGFβ^52^, and TSG6^53^, which are upregulated in tumors with poor prognosis, increased in the DH82 co-cultured group, suggesting that these factors may affect the anti-cancer drug resistance of canine MGT cells.

Furthermore, TAMs induce doxorubicin resistance in breast cancer by upregulating IL-10 via the IL-10/IL10-receptor/STAT3/Bcl-2 signaling pathway^48^. When STAT3 is inhibited in tumor cells, the expression of Bcl-2, which is an anti-apoptotic gene, is reduced, and the apoptotic caspase 3 pathway is activated. Cyclin D1, which is related to tumor cell proliferation, also decreases with STAT3 inhibition^54^. In a previous study, as DH82 were treated with doxorubicin, the expression of IL-10 and VEGF, which are associated M2 polarized macrophage, increased (Supplementary Fig. 1). When canine MGT cells and DH82 were co-cultured, we confirmed that apoptosis was reduced in canine MGT cells, and in this regard, procaspase 3 expression was increased in canine MGT cells under doxorubicin. We also investigated the effect of DH82 on the cell cycle of the canine MGT cells, confirming that doxorubicin induces G2/M phase cell cycle arrest, and cyclin D1 is involved^55^. In the DH82 co-cultured groups, G2/M phase cell cycle arrest and cyclin D1 expression decreased, suggesting that DH82 influenced doxorubicin-induced cell cycle arrest in canine MGT cells. Based on these results, it is thought that crosstalk between DH82 and canine MGT cells causes resistance to anti-cancer drugs.

In conclusion, canine MGT cells and DH82 hybrid spheroid model were constructed. The hybrid spheroid model mimics the TME by reflecting the cross talk of canine MGT cells and DH82 under hypoxia. The hybrid spheroid model can be applied to tumor and anti-cancer drug research in the future, and inhibition of macrophage chemoresistance can be considered a key factor in future anti-cancer treatment.

## Methods

### Cell culture

Canine malignant mammary gland tumor cell lines (CIPp, CIPm) were obtained from the Department of Veterinary Clinicopathology, Seoul National University (SNU). CIPp and CIPm cells were derived from primary and metastatic canine MGTs. Both cell lines were maintained in Roswell Park Memorial Institute (RPMI) 1640 medium (Welgene, Gyeong-San, Republic of Korea) with 10% fetal bovine serum (FBS; Gibco, Billings, MT, USA) and 1% solution of 10,000 units/ml penicillin (Sigma-Aldrich, St. Louis, MO, USA) and 100 µg/ml streptomycin (Sigma-Aldrich) in 5% CO_2_ at 37 °C. Canine macrophage cell line DH82 was purchased from ATCC (ATCC number: CRL-10389, Manassas, VA, USA) and cultured in Dulbecco’s modified Eagle DMEM medium (Welgene) with 10% FBS and 1% penicillin–streptomycin (PS) in 5% CO_2_ at 37 °C. The medium was replaced every 2–3 days. The cells were subcultured until their confluency reached 80–90%.

### Spheroid formation

Canine MGT cells and DH82 were seeded with a total of 2 × 10^5^ cells per well in a Stem FIT 3D cell culture dish (c253000, MicroFIT, Inc., Ha-Nam, Republic of Korea). The cells were incubated in RPMI 1640 medium with 20% FBS and 1% PS in 5% CO_2_ at 37 °C. To construct the hybrid spheroid model, canine MGT cells and DH82 were mixed at different ratios (1:0, 3:1, 1:1, 1:3, and 0:1). Spheroids were estimated every 12 h using an inverted microscope (Olympus CKX53, Olympus, Tokyo, Japan) at 4× magnification. Spheroid areas were measured using ImageJ software (National Institutes of Health, USA, http://imagej.nih.gov/ij/). The medium was changed every 1–2 days.

### Histological analysis of spheroids

Following culturing for 36 h, spheroids were obtained by pipetting carefully. Broken spheroids were excluded. The spheroids were washed thrice with PBS and fixed with neutral buffered 10% formalin with overnight at 4 °C. Fixed spheroids were prepared as blocks using Histogel (Epredia, Kalamazoo, MI, USA). These spheroid blocks were embedded in paraffin, sectioned, and stained with hematoxylin (Thermo Fisher Scientific, Waltham, MA, USA) and Eosin Y (Thermo Fisher Scientific).

### Immunofluorescence analysis using whole spheroid staining

To confirm macrophage polarization, hybrid spheroids (3:1 ratio of canine MGT cells to DH82) were cultured for 36 h. The spheroids were washed thrice with cold DPBS (Welgene) and fixed with neutral buffered 10% formalin with overnight at 4 °C. The fixed spheroids were washed thrice with 0.1% Triton X (Sigma-Aldrich) in DPBS (0.1% PBSTX) and permeabilized with 0.5% triton X in DPBS (0.5% PBSTX) for 2 h at room temperature (RT). After permeabilization, spheroids were washed thrice with 0.1% PBSTX and blocked with 2% bovine serum albumin (BSA; Sigma-Aldrich) in 0.1% PBSTX for 1 h at RT. The spheroids were incubated overnight at 4 °C with fluorescein isothiocyanate (FITC) conjugated mouse anti-CD206 (1:100; BioLegend, San Diego, CA, USA) and phycoerythrin (PE) conjugated mouse anti-CD80 (1:100; BioLegend). After incubation, the spheroids were washed thrice with DPBS and mounted in Antifade mounting medium with 4′,6-diamidino-2-phenylindole (DAPI; VECTASHIELD Vector Laboratories, Burlingame, CA, USA). The spheroids were analyzed by confocal laser scanning microscopy (CLSM) (LSM 710; Zeiss, Oberkochen, Germany).

Furthermore, to confirm hypoxic conditions, canine MGT cells alone spheroids were cultured for 36 h, and then DH82 was placed in wells including spheroids for infiltration. The infiltrated spheroids were incubated with pimonidazole (Hypoxyprobe RedAPC kit, Hypoxyprobe, Inc., Burlington, MA, USA) for 2 h, followed by serial staining with an antibody against pimonidazole and FITC conjugated mouse anti-CD11b (1:100; BioLegend). Hypoxic cell area was measured with Image J.

### Anti-cancer drug treatment

Doxorubicin (DOX; Korea United Pharm, Inc., Cheong-Ju, Republic of Korea) was dissolved in tertiary distilled water and stored at 4 °C up to 2 weeks. Canine MGT cells and DH82 cells were treated with 0, 0.18, 0.37, 0.92 µM of DOX.

### Cell viability assay

Canine MGT and DH82 cells were seeded at 1 × 10^4^ cells/well in 96 well plate and incubated overnight at 37 °C. Cells were treated with different concentrations of DOX for 24, 48, and 72 h. After incubation, the cells were incubated with the Cell Count Kit (CCK) solution (Dong-in Biotech, Seoul, Republic of Korea) for 1 h at 37 °C, and the optical density (OD) was measured at 450 nm using a spectrophotometer (Epoch Microplate spectrophotometer, BioTek Instruments, Winooski, VT, USA).

To evaluate the effect of anti-cancer drug resistance when cultured with macrophages, CIPp and CIPm were seeded at 1 × 10^5^ cells/well in 24 well plate. DH82 cells were seeded at 0.4-µm pore-sized Transwell inserts (SPL Life Science, Po-Cheon, Republic of Korea) at a 3:1 ratio of canine MGT cells: DH82. After overnight incubation, the canine MGT cells and DH82 were co-cultured for 48 h at 37 °C with 0, 0.18, 0.37 µM of DOX and measured cell viability using CCK solution.

### Quantitative reverse transcription polymerase chain reaction measurement

To confirm mRNA expression level changes according to hypoxia conditions in 2D culture and spheroid culture, canine MGT cells were seeded at 5 × 10^4^ cells/well in 6 well plate and 2 × 10^5^ cells per well in a Stem FIT 3D cell culture dish and cultured for 48 h at 37 °C. In addition, to confirm the change in mRNA expression level according to DH82 co-culture, canine MGT cells were seeded at 5 × 10^5^ cells/well in 6 well plate and DH82 were seeded at 5 × 10^4^ cells/well in 0.4-µm pore-sized Transwell inserts (SPL Life Science). After overnight incubation, the canine MGT cell and DH82 were co-cultured and treated with 0, 0.18, and 0.37 µM concentration of DOX for 48 h at 37 °C. Total RNA was extracted using the Easy-BLUE Total RNA Extraction kit (iNtRON Biotechnology, Seong-Nam, Republic of Korea), according to the manufacturer’s protocol. The total RNA concentration and purity of each sample were measured using a spectrometer (NanoPhotometer, Implen, Westlake Village, CA, USA). The cDNA of each sample was synthesized using CellScript All-in-One 5× 1st cDNA Strand Synthesis Master Mix (CellSafe, Yong-In, Republic of Korea). Using AMPIGENE qPCR Green Mix Hi-ROX with SYBR Green dye (Enzo Life Sciences, Farmingdale, NY, USA), the samples were evaluated with 1 µL of cDNA and 400 nM of each forward and reverse primer (BIONICS, Seoul, Republic of Korea). Gene expression levels were normalized to that of glyceraldehyde 3-phosphate dehydrogenase (GAPDH). Primer sequences used in this study are listed in Table 1.

**Table 1:** Primer sequences.

Gene	Forward (5′ > 3′)	Reverse (5′ > 3′)	References
GAPDH	TATGACGACATCAAGAAGGTAGTGA	GTAGCCAAATTCATTGTCATACCAG	^56^
COX2	GCCTTACCCAGTTTGTGGAA	AGCCTAAAGCGTTTGCGATA	^57^
HIF-1a	ATGATGGTGACATGATTTACATTTCT	GTATTCTGCTCTTTACCCTTTTTCAC	^56^
VEGF	GAATGCAGACCAAAGAAAGATAGAG	GATCTTGTACAAACAAATGCTTTCTC	^56^
TGF-b	CTCAGTGCCCACTGTTCCTG	TCCGTGGAGCTGAAGCAGTA	^58^
TSG-6	TCCGTCTTAATAGGAGTGAAAGATG	AGATTTAAAAATTCGCTTTGGATCT	^59^
MRP1	CGTGACCGTCGACAAGAACA	CACGATGCTGATGACCA	^60^
P-gp	CTATGCCAAAGCCAAAGTATC	GAGGGCTGTAGCTGTCAATC	^60^

### Apoptosis analysis

Canine MGT cells were seeded at 5 × 10^5^ cells/well in 6 well plate and DH82 were seeded at 5 × 10^4^ cells/well in 0.4-µm pore-sized Transwell inserts (SPL Life Science). After overnight incubation, the canine MGT cell and DH82 were co-cultured and treated with 0, 0.18, 0.37 and 0.92 µM concentration of DOX for 48 h at 37 °C. After DOX treatment, the canine MGT cells were harvested and washed thrice with cold DPBS. An Annexin V-FITC apoptotic detection kit (Enzo Life Science) was used to detect apoptotic cells according to the manufacturer’s protocol. The cells were resuspended in 1× binding buffer and stained with Annexin V-FITC and PI (dilution 1:20) for 15 min at RT in the dark. Flow cytometry was performed within 1 h using FACS Aria II (BD Biosciences, Franlkin Lakes, NJ, USA) and the results were analyzed using FlowJo software (BD Biosciences).

### Cell cycle analysis

Canine MGT cells were seeded at 5 × 10^5^ cells/well in 6 well plate and DH82 were seeded at 5 × 10^4^ cells/well in 0.4-µm pore-sized Transwell inserts (SPL Life Science). After overnight incubation, the canine MGT cell and DH82 were co-cultured and treated with 0, 0.18, 0.37 and 0.92 µM concentration of DOX for 48 h at 37 °C. After DOX treatment, the canine MGT cells were harvested and washed thrice with cold DPBS. The cells were fixed with cold 70% ethanol for minimum 2 h at − 20 °C. After fixation, the cells were washed with DPBS and stained with propidium iodine/RNase buffer (BD Biosciences) for 15 min at RT in the dark. The samples were analyzed within 1 h using FACS Aria II (BD Biosciences) and the results were analyzed using FlowJo software (BD Biosciences) with waston pragmatic model.

### Western blot analysis

To confirm mRNA expression level changes according to hypoxia conditions in 2D culture and spheroid culture, canine MGT cells were seeded at 5 × 10^4^ cells/well in six well plate and 2 × 10^5^ cells per well in a Stem FIT 3D cell culture dish and cultured for 48 h at 37 °C. In addition, to confirm the change in mRNA expression level according to DH82 co-culture, canine MGT cells were seeded at 5 × 10^5^ cells/well in 6 well plate and DH82 were seeded at 5 × 10^4^ cells/well in 0.4-µm pore-sized Transwell inserts (SPL Life Science). After overnight incubation, the canine MGT cell and DH82 were co-cultured and treated with 0, 0.18, and 0.37 µM concentration of DOX for 48 h at 37 °C. The total protein content was extracted from canine MGT cells using PRO-PREP Extraction Solution (iNtRON Biotechnology) according to the manufacturer’s protocol. The protein concentration of each sample was determined using the DC Protein Assay Kit (Bio-Rad, Hercules, CA, USA). For protein expression analysis, 15 µg protein sample was loaded and separated by 10% sodium dodecyl sulfate–polyacrylamide gel electrophoresis (SDS-PAGE). Proteins were transferred to polyvinylidene difluoride membranes (EMD Millipore, Burlington, MA, USA). The membranes were blocked with 5% skim milk in Tris-buffered saline and incubated with primary antibodies against cyclinD1(1:1000; Cell Signaling Technology, Danvers, MA, USA), caspase-3(1:1000; Cell Signaling Technology), and β-actin (1:1000, Santa Cruz Biotechnology, Dallas, TX, USA) for overnight at 4 °C. After incubation, the membranes were washed and incubated with goat anti-mouse horseradish peroxidase-labeled secondary antibody (Bethyl Laboratories, Montgomery, TX, USA) or goat anti-rabbit horseradish peroxidase-labeled secondary antibody (Enzo Life Sciences) at RT for 1 h. Immunoreactive bands were detected by chemiluminescence (Advansta, San Jose, CA, USA). The bands were imaged using ImageQuant Las4000 mini (GE Healthcare Life Sciences, Chicago, IL, USA) and normalized to β-actin levels.

### Statistical analysis

Each experiment was performed at least thrice. GraphPad Prism (version 9.3.1) software (GraphPad Software, San Diego, CA, USA) was used for statistical analyses. The data were analyzed using Student’s t-test and one-way analysis of variance (ANOVA) followed by Tukey’s multiple comparisons test. Results are presented as mean ± standard deviation (SD). Differences with a value of p < 0.05 were considered as statistically significant.

## Supplementary Information

Supplementary Figures.

## Data Availability

The data that support the findings of this study are available from the corresponding author upon reasonable request.
