# Supplementary material for: Macrophage induces anti-cancer drug resistance in canine mammary gland tumor spheroid
Source: Sci Rep. 2023 Jun 27;13:10394. doi: 10.1038/s41598-023-37311-w (PMC10300191; doi:10.1038/s41598-023-37311-w)
Supplement: Supplementary file 1 — Supplementary Figures. [file 41598_2023_37311_MOESM1_ESM.pdf]

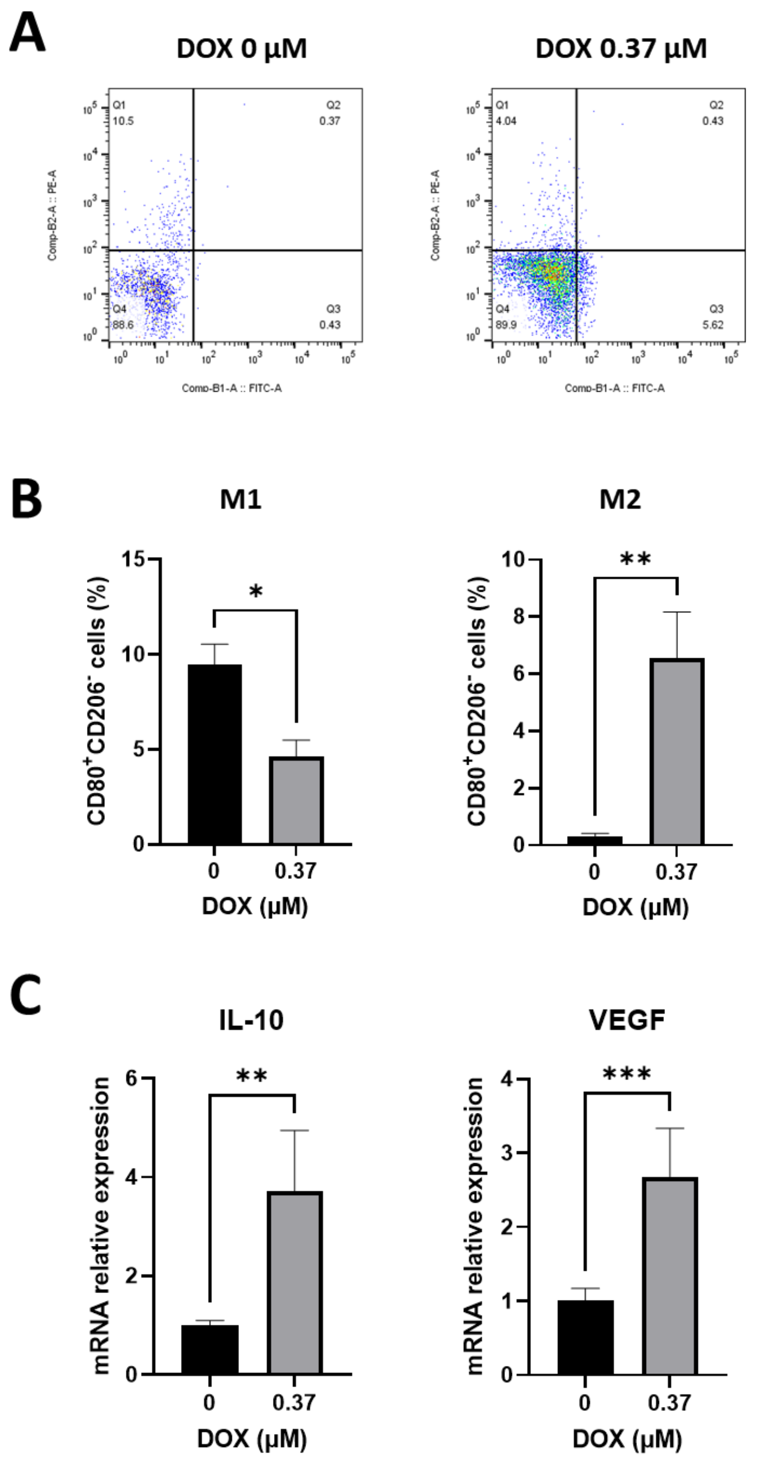

Supplementary figure 1. Doxorubicin induces cytokine overexpression associated with M2 polarization in canine macrophage. DH82 treated 0.37 μM of doxorubicin for 48 hours. A) DH82 stained with FITC conjugated anti-CD206 and PE conjugated anti-CD80 antibody and measured cell distribution by flow cytometry. B) Percentage of CD80+CD206-(M1) cells was decreased and percentage of CD80-CD206+(M2) cells was increased under doxorubicin. C) IL-10 and VEGF increased in DH82 under doxorubicin. The results are presented as the mean ± SD of triplicate samples of three independent experiments. **$p < 0.01$ and ***$p < 0.001$, as determined by Student's t-tests.

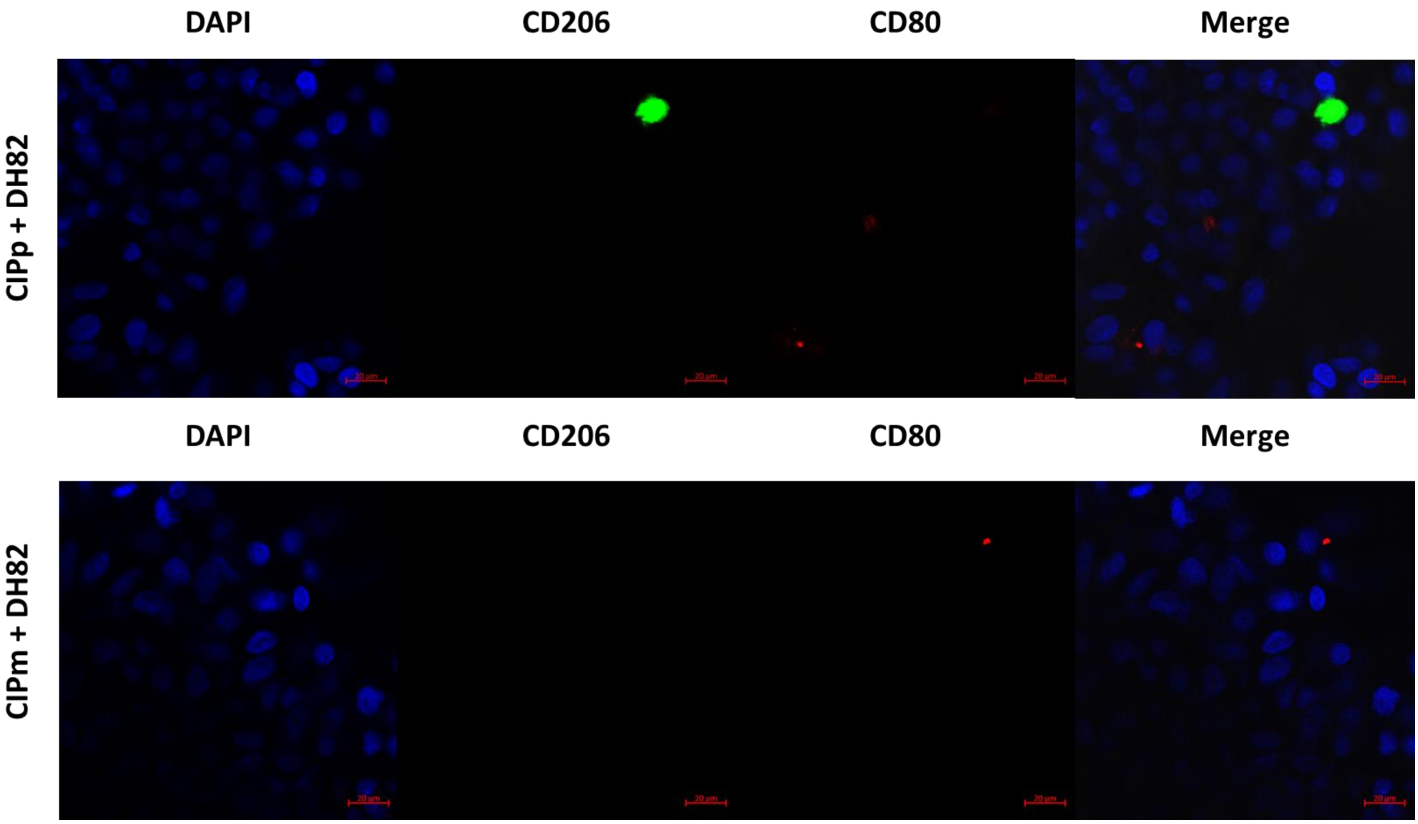

Supplementary figure 2. High magnification Z stack images of figure 1D and 1G (x50; red bar = 20 µm).

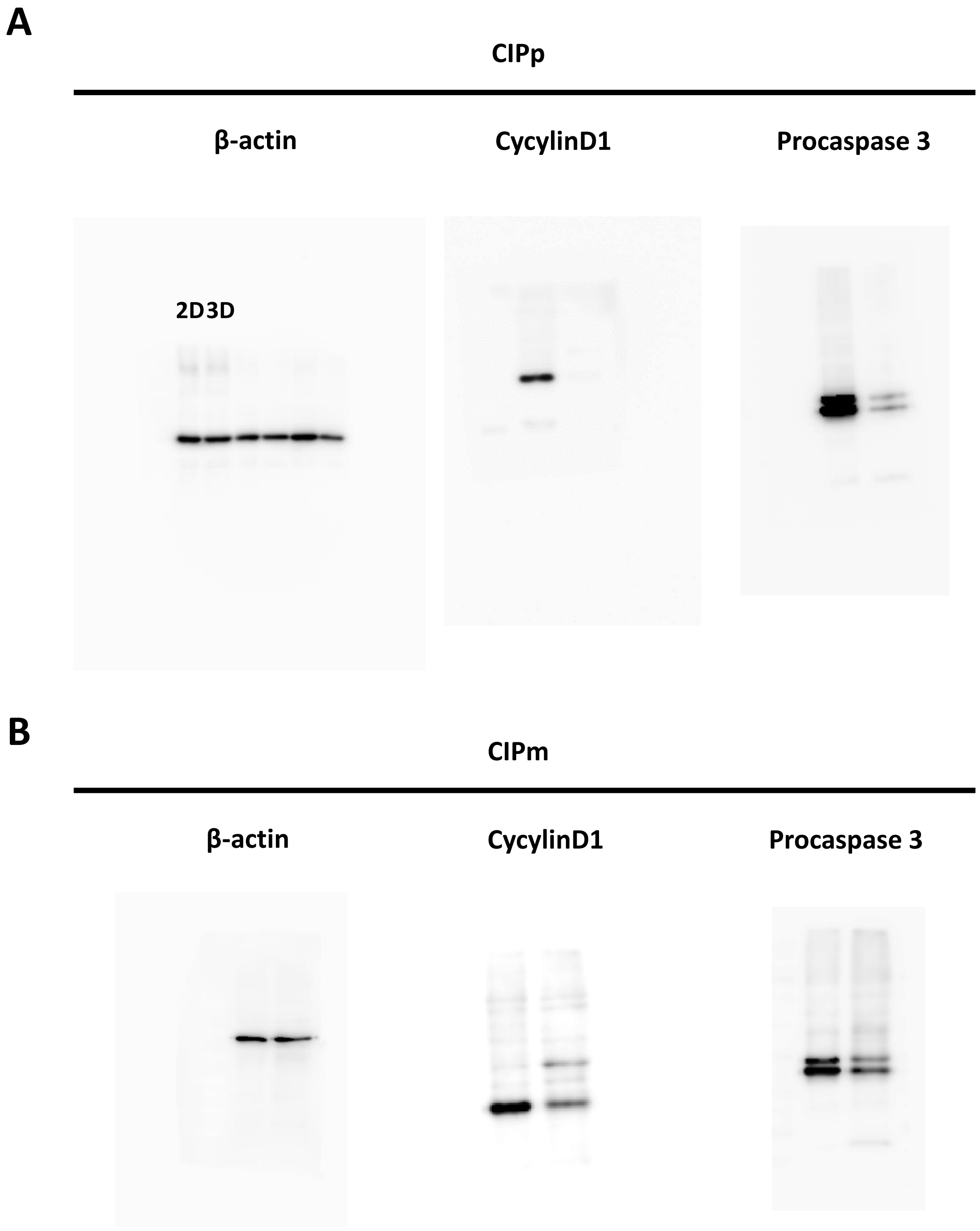

Supplementary figure 3. Western blot original images of figure 2B.

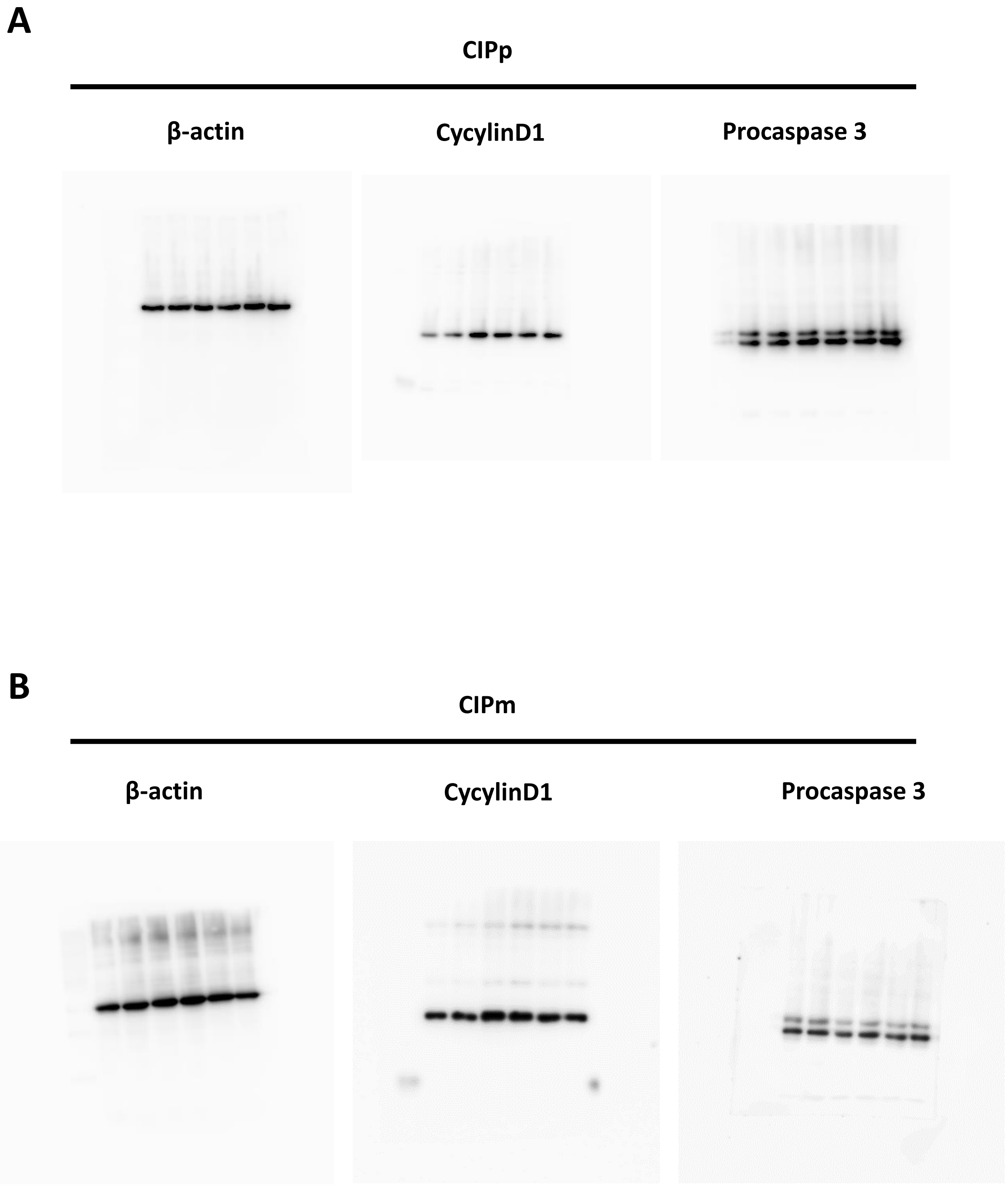

Supplementary figure 4. Western blot original images of figure 5A.
